# Supplementary material for: A novel rhesus macaque model of Huntington’s disease recapitulates key neuropathological changes along with motor and cognitive decline
Source: eLife. 2022 Oct 7;11:e77568. doi: 10.7554/eLife.77568 (PMC9545527; doi:10.7554/eLife.77568)
Supplement: Supplementary file 1. — *p < 0.05, **p < 0.01, ***p < 0.001. [file elife-77568-supp1.docx]

**Supplementary file 1**

| Group Comparison | Timepoint | T-statistic | df | p-value |
| --- | --- | --- | --- | --- |
| 85Q vs Buffer | 3m | -2.048 | 8 | 0.037* |
|  | 6m | -3.652 | 8 | 0.003** |
|  | 9m | -5.762 | 8 | 0.00021** |
|  | 14m | -6.045 | 8 | 0.00015** |
|  | 20m | -3.392 | 8 | 0.005** |
|  | 30m | -2.657 | 7 | 0.016* |
| 85Q vs 10Q | 3m | -1.188 | 9 | 0.133 |
|  | 6m | -1.981 | 9 | 0.039* |
|  | 9m | -3.196 | 9 | 0.005** |
|  | 14m | -4.887 | 9 | 0.0004*** |
|  | 20m | -2.194 | 9 | 0.028* |
|  | 30m | -1.965 | 8 | 0.043* |
| Buffer vs 10Q | 3m | -1.137 | 7 | 0.146 |
|  | 6m | -2.426 | 7 | 0.023* |
|  | 9m | -1.483 | 7 | 0.091 |
|  | 14m | -1.974 | 7 | 0.045* |
|  | 20m | -1.033 | 7 | 0.168 |
|  | 30m | -1.694 | 7 | 0.067 |

**Table S1.** Planned group comparisons for 3-Choice Spatial Delayed Response (SDR) task using one-tailed Independent Sample T-tests at each timepoint. *p<0.05, **p<0.01, ***p<0.001
