# Supplementary material for: A novel rhesus macaque model of Huntington’s disease recapitulates key neuropathological changes along with motor and cognitive decline
Source: eLife. 2022 Oct 7;11:e77568. doi: 10.7554/eLife.77568 (PMC9545527; doi:10.7554/eLife.77568)
Supplement: Supplementary file 2. — *p < 0.05, **p < 0.01, ***p < 0.001. [file elife-77568-supp2.docx]

**Table Supplement 2**

| Group Comparison | Timepoint | T-statistic | df | p-value |
| --- | --- | --- | --- | --- |
| 85Q vs Buffer | 3m | 2.512 | 9 | 0.017* |
|  | 6m | 4.198 | 9 | 0.001** |
|  | 9m | 5.316 | 9 | 0.0002*** |
|  | 14m | 3.057 | 9 | 0.007** |
|  | 20m | 3.324 | 9 | 0.004** |
|  | 30m | 3.027 | 8 | 0.008*** |
| 85Q vs 10Q | 3m | 1.589 | 10 | 0.072 |
|  | 6m | 1.986 | 10 | 0.038* |
|  | 9m | 3.179 | 10 | 0.005** |
|  | 14m | 1.597 | 10 | 0.071 |
|  | 20m | 1.996 | 10 | 0.037* |
|  | 30m | 1.587 | 9 | 0.073 |
| Buffer vs 10Q | 3m | 1.631 | 9 | 0.069 |
|  | 6m | 2.203 | 9 | 0.028* |
|  | 9m | 1.609 | 9 | 0.071 |
|  | 14m | 2.733 | 9 | 0.012* |
|  | 20m | 1.833 | 9 | 0.05* |
|  | 30m | 3.151 | 9 | 0.006** |

**Table S2.** Planned group comparisons for Lifesaver Retrieval Latencies using one-tailed Independent Sample T-tests at each timepoint. *p<0.05, **p<0.01, ***p<0.001
