# Supplementary material for: A novel rhesus macaque model of Huntington’s disease recapitulates key neuropathological changes along with motor and cognitive decline
Source: eLife. 2022 Oct 7;11:e77568. doi: 10.7554/eLife.77568 (PMC9545527; doi:10.7554/eLife.77568)
Supplement: Supplementary file 3. — Behaviors were scored cage-side by trained observers, blinded to treatment condition, during 30- to 45-min focal observations. Scores were summed across categories to generate a total NRS score. Higher scores indicate more severe phenotypes. [file elife-77568-supp3.docx]

**Supplementary file 3**

| Phenotype | Score Description |
| --- | --- |
| Homecage ambulation | 0-normal, uses all 4 limbs smoothly and symmetrically 1-minimally slow, ambulating around cage 2-moderately slow, ambulating with difficulty 3-marked slowing, no ambulation |
| Balance | 0- normal 1- slight inability to maintain balance while sitting and ambulating 2- moderate inability to maintain balance, requires cage support 3-recumbent |
| Ocular pursuit  *(Horizontal and vertical)* | 0-complete 1-jerky 2-incomplete range 3-cannot pursue |
| Treat retrieval  *(Right and left forelimbs)* | 0-normal 1-minimally slow, treat retrieved & eaten 2-markedly slow, treat retrieved & eaten 3-marked slowing, treat not retrieved |
| Weight bearing  *(Right and left hindlimbs)* | 0-normal, capable of bearing full weight 1-can bear full weight only some of the time 2-can bear partial weight some of the time 3-incapable, cannot bear any weight |
| Dystonia  *(Orofacial, trunk and limbs)* | 0-absent 1-slight/intermittent 2-moderate/common 3-marked/prolonged |
| Chorea  *(Orofacial, trunk and limbs)* | 0-absent 1-slight/intermittent 2-moderate/common 3-marked/prolonged |
| Bradykinesia  *(Orofacial, trunk and limbs)* | 0-absent 1-slight/intermittent 2-moderate/common 3-marked/prolonged |
| Ataxia  *(Orofacial, trunk and limbs)* | 0-absent 1-slight/intermittent 2-moderate/common 3-marked/prolonged |
| Tremor  *(Orofacial, trunk and limbs)* | 0-absent 1-slight/intermittent 2-moderate/common 3-marked/prolonged |
| Dysmetria  *(Orofacial, trunk and limbs)* | 0-absent 1-slight/intermittent 2-moderate/common 3-marked/prolonged |

**Table S3. Neurological Rating Scale for Nonhuman Primates**. Behaviors were scored cage-side by trained observers, blinded to treatment condition, during 30–45-minute focal observations. Scores were summed across categories to generate a total NRS score. Higher scores indicate more severe phenotypes.
