# Supplementary material for: A novel rhesus macaque model of Huntington’s disease recapitulates key neuropathological changes along with motor and cognitive decline
Source: eLife. 2022 Oct 7;11:e77568. doi: 10.7554/eLife.77568 (PMC9545527; doi:10.7554/eLife.77568)
Supplement: Supplementary file 4. — *p < 0.05, **p < 0.01, ***p < 0.001. [file elife-77568-supp4.docx]

**Table Supplement 4**

| **Group Comparison** | **Timepoint** | **T-statistic** | **df** | **p-value** |
| --- | --- | --- | --- | --- |
| 85Q vs Buffer | 0.5m | -1.809 | 9 | 0.052 |
|  | 1m | -1.520 | 9 | 0.088 |
|  | 2m | 0.734 | 9 | 0.241 |
|  | 3m | 2.555 | 9 | 0.016* |
|  | 4m | 2.352 | 9 | 0.022* |
|  | 5m | 3.371 | 9 | 0.004** |
|  | 6m | 4.629 | 9 | 6.26E-4*** |
|  | 7m | 2.961 | 9 | 0.008** |
|  | 8m | 7.151 | 9 | 2.72E-05*** |
|  | 9m | 3.160 | 9 | 0.006** |
|  | 10m | 4.931 | 9 | 4.11E-4*** |
|  | 11m | 5.149 | 9 | 3.02E-4*** |
|  | 12m | 8.613 | 9 | 6.11E-06 |
|  | 13m | 5.056 | 9 | 3.42E-4*** |
|  | 14m | 6.945 | 9 | 3.36E-05*** |
|  | 15m | 8.906 | 9 | 4.65E-06*** |
|  | 16m | 5.002 | 9 | 3.68E-4*** |
|  | 17m | 5.859 | 9 | 1.21E-4*** |
|  | 18m | 12.043 | 9 | 3.74E-7*** |
|  | 19m | 11.687 | 9 | 4.82E-7*** |
|  | 20m | 6.182 | 9 | 8.10E-5*** |
|  | 21m | 11.442 | 9 | 5.77E-7*** |
|  | 22m | 10.011 | 9 | 2.00E-6*** |
|  | 23m | 8.649 | 9 | 6.00E-6*** |
|  | 24m | 15.207 | 9 | 5.01E-8*** |
|  | 25m | 8.277 | 9 | 8.42E-6*** |
|  | 26m | 24.000 | 8 | 4.84E-9*** |
|  | 27m | 12.944 | 8 | 6.01E-7*** |
|  | 28m | 5.421 | 8 | 3.15E-4*** |
|  | 29m | 5.687 | 8 | 2.31E-4*** |
|  | 30m | 10.633 | 8 | 2.68E-6*** |
| 85Q vs 10Q | 0.5m | 1 | 10 | 0.170 |
|  | 1m | 1 | 10 | 0.170 |
|  | 2m | 1.861 | 10 | 0.0462* |
|  | 3m | 2.996 | 10 | 0.0067** |
|  | 4m | 2.785 | 10 | 0.009** |
|  | 5m | 3.478 | 10 | 0.003** |
|  | 6m | 5.082 | 10 | 2.38E-4*** |
|  | 7m | 3.411 | 10 | 0.003** |
|  | 8m | 6.742 | 10 | 2.50E-5*** |
|  | 9m | 3.034 | 10 | 0.006** |
|  | 10m | 5.117 | 10 | 2.26E-4*** |
|  | 11m | 5.757 | 10 | 9.2E-5*** |
|  | 12m | 9.396 | 10 | 1.40E-6*** |
|  | 13m | 5.371 | 10 | 1.57E-4*** |
|  | 14m | 6.103 | 10 | 5.77E-5*** |
|  | 15m | 7.590 | 10 | 9.31E-6*** |
|  | 16m | 6.277 | 10 | 4.59E-5*** |
|  | 17m | 7.590 | 10 | 9.31E-6*** |
|  | 18m | 13.348 | 10 | 5.33E-8*** |
|  | 19m | 11.364 | 10 | 2.43E-7*** |
|  | 20m | 5.813 | 10 | 8.94E-5*** |
|  | 21m | 6.063 | 10 | 6.07E-5*** |
|  | 22m | 6.708 | 10 | 2.66E-5*** |
|  | 23m | 7.604 | 10 | 9.16E-6*** |
|  | 24m | 6.934 | 10 | 2.01E-5*** |
|  | 25m | 5.168 | 10 | 2.10E-4*** |
|  | 26m | 13.473 | 9 | 1.43E-7*** |
|  | 27m | 10.062 | 9 | 1.70E-6*** |
|  | 28m | 6.728 | 9 | 4.29E-5*** |
|  | 29m | 5.513 | 9 | 1.87E-4*** |
|  | 30m | 8.612 | 9 | 6.11E-6*** |
| Buffer vs 10Q | 0.5m | -2.018 | 9 | 0.037* |
|  | 1m | -1.764 | 9 | 0.056 |
|  | 2m | -1.543 | 9 | 0.079 |
|  | 3m | -0.905 | 9 | 0.195 |
|  | 4m | -0.905 | 9 | 0.195 |
|  | 5m | 0.00 | 9 | 0.500 |
|  | 6m | -0.905 | 9 | 0.195 |
|  | 7m | -0.905 | 9 | 0.195 |
|  | 8m | 0.000 | 9 | 0.500 |
|  | 9m | 0.267 | 9 | 0.398 |
|  | 10m | 0.00 | 9 | 0.500 |
|  | 11m | -0.408 | 9 | 0.347 |
|  | 12m | -0.905 | 9 | 0.195 |
|  | 13m | 0.00 | 9 | 0.500 |
|  | 14m | 0.905 | 9 | 0.195 |
|  | 15m | 0.267 | 9 | 0.398 |
|  | 16m | -1.398 | 9 | 0.098 |
|  | 17m | -0.811 | 9 | 0.219 |
|  | 18m | -0.129 | 9 | 0.450 |
|  | 19m | 0.00 | 9 | 0.500 |
|  | 20m | 0.267 | 9 | 0.398 |
|  | 21m | 1.945 | 9 | 0.042* |
|  | 22m | 1.430 | 9 | 0.093 |
|  | 23m | 1.152 | 9 | 0.139 |
|  | 24m | 1.752 | 9 | 0.057 |
|  | 25m | 1.036 | 9 | 0.164 |
|  | 26m | 0.278 | 9 | 0.398 |
|  | 27m | 0.376 | 9 | 0.358 |
|  | 28m | -1.969 | 9 | 0.040* |
|  | 29m | 0.905 | 9 | 0.195 |
|  | 30m | 1.809 | 9 | 0.052 |

**Table S4.** Planned Group Comparisons in Monthly NRS Scores using one-tailed Independent Sample T-tests at each timepoint. *p<0.05, **p<0.01, ***p<0.001
