# Supplementary material for: A novel rhesus macaque model of Huntington’s disease recapitulates key neuropathological changes along with motor and cognitive decline
Source: eLife. 2022 Oct 7;11:e77568. doi: 10.7554/eLife.77568 (PMC9545527; doi:10.7554/eLife.77568)
Supplement: Supplementary file 5. — *p < 0.05, **p <0.01. [file elife-77568-supp5.docx]

**Supplementary file 5**

| Group Comparison | Timepoint | T-statistic | df | p-value |
| --- | --- | --- | --- | --- |
| Buffer | 3m | -1.500 | 4 | 0.104 |
|  | 6m | -1.633 | 4 | 0.089 |
|  | 9m | -1.633 | 4 | 0.089 |
|  | 14m | -1.606 | 4 | 0.092 |
|  | 20m | -1.633 | 4 | 0.089 |
| 10Q | 3m | -1.000 | 5 | 0.182 |
|  | 6m | -1.000 | 5 | 0.182 |
|  | 9m | -1.185 | 5 | 0.145 |
|  | 14m | -0.791 | 5 | 0.233 |
|  | 20m | -0.598 | 5 | 0.288 |
| 85Q | 3m | -3.050 | 5 | 0.014* |
|  | 6m | -3.512 | 5 | 0.009** |
|  | 9m | -4.540 | 5 | 0.003** |
|  | 14m | -1.784 | 5 | 0.067 |
|  | 20m | -2.154 | 5 | 0.042* |

**Table S5.** Planned Comparisons for Pre- vs Post-Apomorphine NRS Scores at each timepoint using one-tailed Paired-Sample T-tests for each group separately. *p<0.05, **p<0.01
